# Predictive value and clinical significance of increased SSAT-1 activity in healthy adults

**DOI:** 10.2144/fsoa-2019-0023

**Published:** 2019-07-01

**Authors:** Paramjit S Tappia, Andrew W Maksymiuk, Daniel S Sitar, Parveen S Akhtar, Nazrina Khatun, Rahnuma Parveen, Rashiduzzaman Ahmed, Rashid B Ahmed, Brian Cheng, Gina Huang, Horacio Bach, Brett Hiebert, Bram Ramjiawan

**Affiliations:** 1 Asper Clinical Research Institute & Office of Clinical Research, St Boniface Hospital, Winnipeg, MB, R2H 2A6, Canada; 2 Cancer Care Manitoba, Winnipeg, MB, R3E 0V9, Canada; 3 Department of Internal Medicine, Rady Faculty of Health Sciences, University of Manitoba, Winnipeg, MB, R3A 1R9, Canada; 4 Department of Pharmacology & Therapeutics, Rady Faculty of Health Sciences, University of Manitoba, Winnipeg, MB, R3E 0T5, Canada; 5 Department of Medical Oncology, National Institute of Cancer Research & Hospital, Mohakhali, Dhaka, Bangladesh; 6 FastBios, Dhaka, Bangladesh; 7 BioMark Diagnostics Inc., Richmond, BC, V6X 2W8, Canada; 8 Division of Infectious Diseases, Faculty of Medicine, University of British Columbia, Vancouver, BC, V5Z 3J5, Canada; 9 Cardiac Sciences Program, Asper Clinical Research Institute, Winnipeg, MB, R2H 2A6, Canada

**Keywords:** amantadine, biomarkers, cancer screening and diagnostics, early detection, SSAT-1

## Abstract

**Aim:**

Spermidine/spermine N^1^-acetyltransferase (SSAT-1) regulates cell growth, proliferation and death. Amantadine is converted by SSAT-1 to acetylamantadine (AA). In our earlier studies, although SSAT-1 was activated in patients with cancer, a number of ostensibly healthy adult volunteers had higher than expected AA concentration. This study was therefore undertaken to examine the outlier group.

**Materials & methods:**

A follow up of urine analysis for AA by liquid chromatography-tandem mass spectrometry as well as clinical assessments and additional blood analyses were conducted.

**Results:**

In some of the outlier controls, higher than expected AA concentration was linked to increased serum carcinoembryonic antigen. Clinical and radiographic assessments revealed underlying abnormalities in other cases that could represent premalignant conditions. Hematology tests revealed elevations in white blood cells and platelets, which are markers of inflammation.

**Conclusion:**

High urine concentration of AA could be used as a simple and useful test for screening of cancer in high-risk populations.

## Lay abstract

This study describes the potential of a novel noninvasive urine test for screening of cancer using a safe and approved drug, amantadine. We have previously measured high urinary concentration of the acetylated form of amantadine in patients diagnosed with cancer. However, higher than expected acetylated amantadine concentration was also measured in some of the healthy adult volunteers. Subsequent clinical assessments revealed that these healthy individuals could have early clinical signs of cancer. This is a simple test, which may serve as a useful tool for routine screening in populations considered at high risk for cancer.

## Graphical abstract

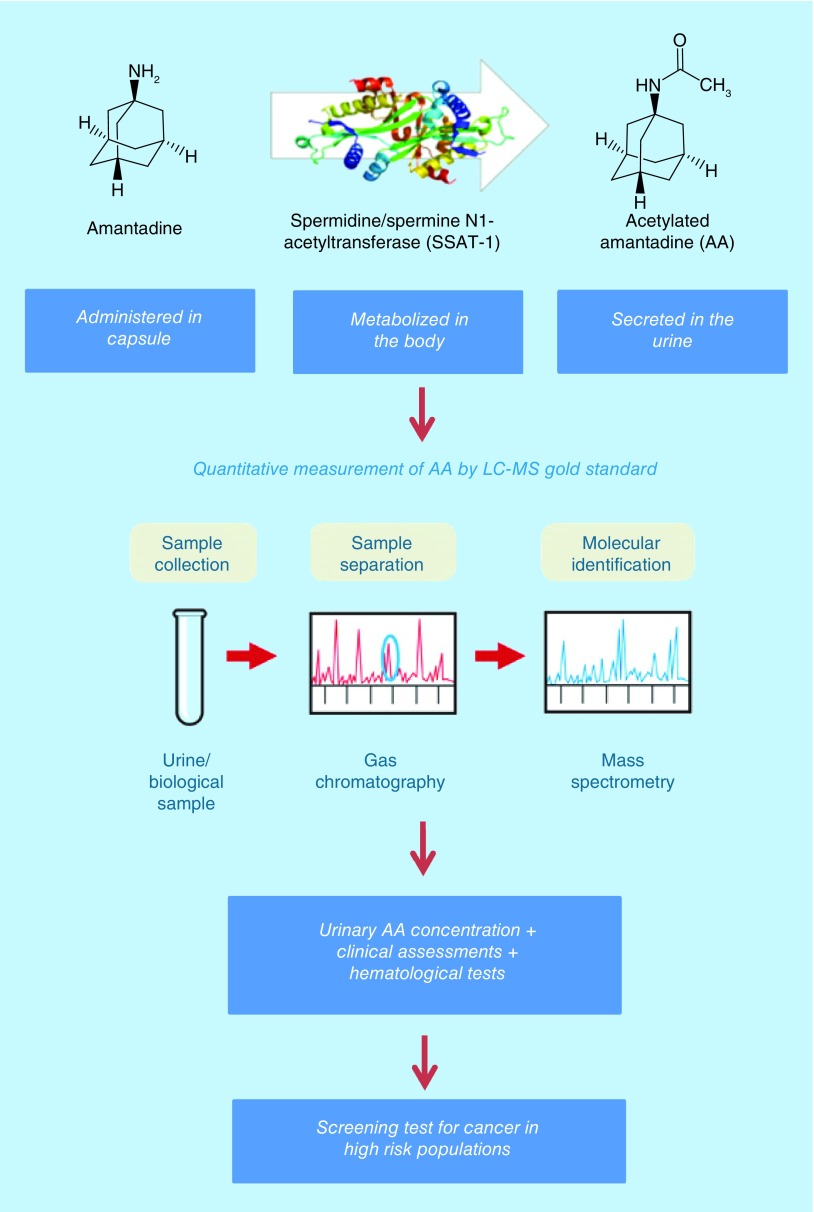

The burden of cancer worldwide, according to the global cancer incidence, mortality and prevalence report (GLOBOCAN 2018) [1], estimated that in 2018 there were 18.1 million new cancer cases (17.0 million excluding nonmelanoma skin cancer) and 9.6 million cancer deaths (9.5 million excluding nonmelanoma skin cancer). In addition, in both men and women combined, lung cancer was the most commonly diagnosed cancer (11.6% of the total cases) and the leading cause of cancer death (18.4% of the total cancer deaths). Other types of cancer with the highest mortality rates are female breast cancer (11.6%), colorectal cancer (9.2%), stomach cancer (8.2%), and liver cancer (8.2%). Of note, lung cancer was the most frequent cancer and the leading cause of cancer death among males, followed by liver and stomach cancer. On the other hand, in women, breast cancer was the most commonly diagnosed cancer as well as the leading cause of cancer death, followed by lung and colorectal cancer. Interestingly, cervical cancer ranks fourth for mortality [1,2]

Early detection and diagnosis of cancer can lead to timely therapeutic/surgical interventions that can increase the chances of survival. Despite intense research and discovery over the years of many candidate biomarkers for cancer screening, only a few have transitioned to routine usage in the clinic [3]. Promising candidates that are in the discovery and developmental mode include circulating tumor cells and cell-free tumor DNA [4,5], miRNAs [6,7], secretome of the gut microbiota [8], exhaled volatile organic compounds, proteomics and metabolomics (liquid biopsy) [9–11]. These face many challenges before routine implementation in the clinical setting [11,12]. In order to introduce a general cancer screening test into practice, the cost and access are important factors that need to be taken into account. Development of a simple, clinically viable, economical, accurate and reproducible screening test would be highly beneficial for the at-risk population.

Polyamines are intimately involved in cell growth, proliferation and cell death [13,14]. Spermidine/spermine N^1^-acetyltransferase-1 (SSAT-1) is a key enzyme in the polyamine metabolic pathway and is known to be upregulated in cancer [15–17]. Amantadine, which is a US FDA-approved antiviral drug and anti-Parkinson’s medication, is a specific substrate of SSAT-1. Amantadine is acetylated by SSAT-1 to produce acetylamantadine (AA) [18], which is a stable end product excreted in urine [18,19]. We have reported the clinical utility of amantadine to detect elevated SSAT-1 activity by measuring increased concentration of AA in the urine of cancer patients [20,21]. In earlier studies, a proportion of the healthy adult controls were deemed to be ‘outliers’ because of higher than expected AA concentrations in the urine. Accordingly, the present study was undertaken to further investigate the outlier group by: performing a follow-up amantadine test; conducting thorough clinical and hematological assessments; and conducting a follow-up health status questionnaire and accessing electronic medical records of these individuals.

## Materials & methods

### Regulatory & institutional review board approvals

Ethics approval was obtained from the University of Manitoba Biomedical Research Ethics Board (Ethics File #s: B2012:063 and B2011:073) prior to study implementation. The study protocol was reviewed and approved by Health Canada (File # 9427-B2749-21C): Notice of Authorization was dated 17 July 2012 and was also listed on the NIH Clinicaltrials.gov website (Identifier: NCT02277938). Appropriate Institutional Review Board (Ministry of Health & Family Welfare, the People’s Republic of Bangladesh) approvals were also obtained (Ethics File # NICRH/Ethics/2017/288). Clinical studies were completed under GCP and GLP conditions in accordance with the standards established by the Canadian Tri-Council Policies.

## Experimental subjects

Healthy controls (n = 40) were recruited by the National Institute of Cancer Research & Hospital, Department of Medical Oncology, Mohakhali, Dhaka, Bangladesh within the local area as part of a study that was being conducted to investigate urinary AA levels in cancer patients [21]. 20 healthy adult controls were also recruited locally at the Asper Clinical Research Institute, St Boniface Hospital, Winnipeg, Canada. All participants provided a signed informed consent for participation. Inclusion criteria were volunteers aged between 18 and 80 years with no history of liver or kidney disease; exclusion criteria were alcohol consumption within 5 days of amantadine ingestion, previous adverse reaction to amantadine and currently pregnant or lactating females. At time of recruitment and participation in the study, none of the healthy control volunteers reported a diagnosis of cancer. After overnight fasting, participants were requested to provide a complete urine collection on the day of the study prior to ingesting amantadine. They then ingested orally 200 mg (2 × 100 mg) amantadine capsules (Mylan-Amantadine, amantadine hydrochloride, USP). Urine was then collected at 2, 4 and 6 h postamantadine ingestion for analysis as previously described [20,21]. Participants that were deemed as outliers were consented for follow-up after obtaining appropriate approvals from the respective institutional review boards.

## Analytical procedures & data cross-validation

Urine was analyzed for AA by established and validated GLP-compliant LC–MS/MS methods using d_3_-AA as the internal standard for quantitation at Biopharmaceuticals Research Inc. (BC, Canada; Study #: BIM-2015-001). Health Canada authorized Biomark AA assay standard under application no: 229838 on 7 October 2014 (Investigational Testing Authorization). The measurement of urinary concentration of AA was conducted as previously described [20,21], Data were cross-validated as described elsewhere [22,23]. The study staff coded the samples and the technician analyzing the biological samples was blinded to participant information.

## Blood analyses & other clinical assessments

Hematological tests, mammogram and ultrasound were conducted using standard procedures at the National Institute of Cancer Research & Hospital, Dhaka, Bangladesh. Serum CEA was measured at Life Labs (ON, Canada) by immunoassay.

## Statistical analysis

Microcal Origin 6 software was used for the calculation of mean values and standard error of the mean (SEM). Box plots were constructed using GraphPad Prism 8.1.

## Results

### Healthy control & cancer patient characteristics

The demographic information of each of the healthy adult participants is shown in Table 1 (Bangladesh site) and Table 2 (Winnipeg site). The mean age (±SEM) of the healthy group at the Bangladesh site (n = 40; 20 male, 20 female) was 52 ± 2 years, whereas the mean age (±SEM) of the healthy group at the Winnipeg site (n = 20; 9 male, 11 female) was 38 ± 3 years. In the Bangladesh cohort, 11/40 (27%) and 2/40 (5%) participants were considered to be overweight (body mass index [BMI] = 25.0–29.9) and obese (BMI ≥30.0), respectively. In the Winnipeg cohort, 10/20 (50%) and 3/20 (15%) were considered to be overweight (BMI = 25.0–29.9) and obese (BMI≥ 30.0), respectively. On the other hand, 6/40 (15%) subjects in the Bangladesh healthy group were deemed to be underweight (BMI <18.5), while only one participant (5%) in the Winnipeg healthy group was determined to be underweight.

**Table 1.** Demographic information on healthy adult volunteers recruited locally at Dhaka (Bangladesh site).

Subject ID	Age (years)	Height (ft; inch)	Weight (lbs)	BMI (kg/m^2^)	Sex (M/F)	Subject ID	Age (years)	Height (ft; inch)	Weight (lbs)	BMI sex (kg/m^2^; M/F)
H01	50	5’5”	132.2	22.0	M	H21	54	5’1”	134.5	25.4 F
H02	27	5’2”	119.1	21.8	F	H22	51	4’11”	119.1	24.1 F
H03	44	4’10”	141.1	29.5	F	H23	55	5’0”	90.4	17.7 F
H04	46	5’2”	156.5	28.6	F	H24	58	5’6”	127.7	20.6 M
H05	43	5’3”	154.3	27.3	F	H25	56	5’6”	132.3	21.4 M
H06	50	5’6”	110.2	17.8	M	H26	57	5’5”	160.9	26.8 M
H07	42	5’11”	178.6	24.9	M	H27	52	5’2”	143.3	26.2 F
H08	29	5’2”	121.3	22.2	F	H28	42	5’1”	152.1	28.7 F
H09	37	5’1”	121.3	22.9	F	H29	52	4’11”	88.2	17.8 F
H10	50	5’1”	108.0	20.4	F	H30	67	5’1”	81.6	15.4 F
H11	56	4’11”	194.0	39.2	F	H31	58	5’5”	141.1	23.5 M
H12	34	5’2”	114.6	21.0	F	H32	50	5’4”	138.9	23.8 M
H13	53	5’3”	123.5	21.9	M	H33	55	5’7”	123.5	19.3 M
H14	48	5’5”	143.3	23.8	M	H34	58	5’8”	152.1	23.1 M
H15	46	5’3”	132.3	22.0	M	H35	59	5’5”	154.3	25.7 M
H16	70	4’9”	121.3	26.3	F	H36	55	5’6”	154.3	24.9 M
H17	38	5’0”	138.9	27.1	F	H37	62	5’8”	138.9	21.1 M
H18	52	4’11”	154.3	31.2	F	H38	55	5’7”	158.7	24.9 M
H19	55	5’2”	83.8	15.3	M	H39	63	5’5”	138.9	23.1 M
H20	75	4’10”	88.2	18.4	F	H40	61	5’4”	154.3	26.5 M

The BMI is defined according to [46]. BMI scale: <18.5 underweight; 18.5–24.9 normal; 25.0–29.9 overweight; >30.0 obese.

BMI: Body mass index; F: Female; M: Male; ’ = Feet; ” = Inches.

**Table 2.** Demographic information on healthy adult volunteers recruited locally at the Asper Clinical Research Institute (Winnipeg site).

Subject ID	Age (years)	BMI (kg/m2)	Weight (lbs)	BMI (kg/m^2^)	Sex (M/F)
BM0001	28	5’7”	136.5	22.0	F
BM0002	24	5’5”	158.6	27.2	F
BM0003	57	5’3”	164.2	29.1	F
BM0004	34	5’7”	177.8	27.8	F
BM0005	49	5’3”	132.0	23.4	F
BM0006	46	5’5”	180.0	30.0	F
BM0007	28	5’3”	158.4	28.1	F
BM0008	49	5’3”	116.5	20.6	F
BM0009	22	5’3”	144.0	25.5	F
BM0010	63	5’3”	158.4	28.1	F
BM0011	25	5’7”	115.7	18.1	F
BM0021	50	5’9”	200.2	29.6	M
BM0022	61	5’9”	181.2	26.8	M
BM0023	51	6’0”	232.4	31.5	M
BM0024	54	6’0”	203.8	27.6	M
BM0025	23	5’5”	140.0	23.3	M
BM0026	43	5’5”	157.0	26.1	M
BM0027	19	5’7”	141.6	22.2	M
BM0028	18	5’8”	149.2	22.7	M
BM0029	18	5’1”	178.4	33.7	M

The BMI is defined according to [46]. BMI scale: <18.5 underweight; 18.5–24.9 normal; 25.0–29.9 overweight; >30.0 obese.

BMI: Body mass index; F: Female; M: Male; ’ = Feet; ” = Inches.

### Delineation of ‘outliers’

**Figure 1.**
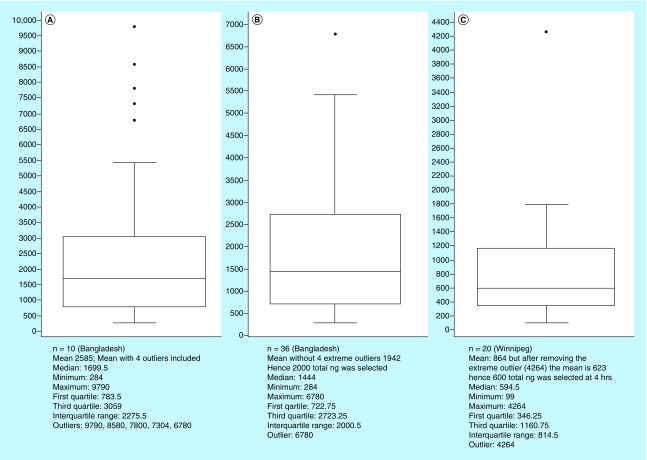
Box plot statistical data for total acetylamantadine amount in healthy volunteers at study sites. Data are presented for Bangladesh **(A & B)** and Winnipeg **(C)** healthy groups. Box plots were constructed to ascertain cutoff points in ostensibly normal individual’s for determination of ‘outliers’ according to the amantadine test.

Figure 1 shows the box plots statistical data with minimum, first quartile, median, third quartile and maximum for AA. Total AA mean excretion at 4 h (ng) in Winnipeg volunteers was 600 ng that provided a tighter assessment after extreme outlier were accounted for in isolating outliers. For the Bangladesh cohort, a 2000 ng at 6 h provided a better cutoff point in selection of outlier definition following management of extreme outliers.

#### Urinary concentrations of AA in healthy adult volunteers (Bangladesh site)

Analysis of the urine samples revealed that in the Bangladesh site there, was a higher than expected concentration (≥24.0 ng/ml) or total amount (>2000 ng) of AA at 6 h in 22/40 (50%) of the healthy adult volunteers (Table 3). The serum concentration of CEA was also determined in the 40 healthy adult volunteers. As shown in Table 4, 18/40 (45%) participants exhibited CEA concentration ≥2.5 ng/ml. However, 11/18 (61%) of the participants were considered outliers because of the overlapping between the results of the AA test combined with the CEA concentrations. An attempt to contact these 11 participants for a follow-up was made with seven favorable responders (H03, H07, H11, H12, H17, H18 and H26). The follow-up was conducted at 6 and 9 months after completion of the initial amantadine test. Interestingly, five out of seven (71%) participants showed an increase in the 2 h urinary AA concentration at 6 months (Table 5) as compared with the concentration observed with the initial amantadine test, which decreased at the 9-month follow-up to near initial values.

**Table 3.** Urinary acetylamantadine concentration in healthy adult volunteers recruited locally at Dhaka (Bangladesh site).

Subject ID	2 h [AA] (ng/ml)	Urine (ml)	Total AA (ng)	4 h [AA] (ng/ml)	Urine (ml)	Total AA (ng)	6 h [AA] (ng/ml)	Urine (ml)	Total AA (ng)
H01^†^	25.4	70	1778	24.4	85	2074	58.2	20	1164
H02	20.1	110	2211	10.8	120	1296	16.9	115	1944
H03^†^	11.9	100	1190	165	100	16,500	156	55	8580
H04^†^	6.0	80	478	65.3	80	5224	32.1	85	2729
H05^†^	49.5	90	4455	83.3	85	7081	178	55	9790
H06^†^	10.5	90	945	26.2	70	1834	83.2	65	5408
H07^†^	11.0	85	935	29.1	90	2619	66.4	60	3984
H08^†^	24.7	55	1359	37.6	80	3008	34.3	90	3087
H09^†^	42.0	100	4200	42.0	70	2940	40.6	100	4060
H10^†^	11.6	75	870	43.3	60	2598	156	50	7800
H11^†^	3.0	130	384	185	35	6475	45.1	60	2706
H12^†^	31.4	70	2198	132	60	7920	91.3	80	7304
H13^†^	6.4	70	446	53.1	65	3452	113	60	6780
H14	21.1	80	1688	19.4	100	1940	19.4	75	1455
H15	7.7	90	697	10.5	70	735	16.2	75	1215
H16	14.6	95	1387	24.7	80	1976	9.4	65	614
H17^†^	9.8	100	984	60.8	100	6080	27.3	90	2457
H18^†^	22.8	100	2280	28.2	90	2538	25.1	90	2259
H19	5.2	120	623	18.0	100	1800	8.1	90	725
H20	14.9	90	1341	24.0	110	2640	8.0	120	959
H21	9.0	75	677	10.2	80	816	4.7	80	378
H22	6.8	60	408	30.9	95	2934	5.2	90	466
H23	4.3	60	256	7.1	60	425	3.6	80	284
H24	7.5	65	488	14.3	100	1430	9.6	75	722
H25	12.8	45	576	11.6	40	464	2.8	110	306
H26^†^	63.3	90	5697	35.8	75	2685	10.5	200	2100
H27	21.5	90	1935	30.7	50	1535	22.6	55	1243
H28	3.2	80	254	80.6	60	484	12.6	115	1449
H29	1.5	110	164	6.1	110	673	13.7	105	1439
H30^†^	2.9	70	204	13.8	55	759	96	30	2880
H31^†^	3.5	120	414	14.7	70	1029	34.6	70	2422
H32^†^	18.0	100	1800	14.5	90	1305	42.5	70	2975
H33^†^	6.3	100	626	34.6	70	2422	52.6	80	4208
H34	47.2	100	4720	2.8	80	227	7.2	75	542
H35	11.3	110	1243	13.9	110	1529	13.5	100	1350
H36	23.1	100	2310	26.7	105	2804	11.5	100	1150
H37	7.7	80	617	13.7	100	1370	15.2	90	1368
H38	1.6	80	127	8.1	75	609	5.6	95	535
H39^†^	22.0	80	1760	24.2	100	2420	24.0	95	2280
H40	7.4	105	781	19.2	85	1632	11.20	85	286

† Identified as an outlier based on total AA amount (ng) at 6 h time point of >2000 ng or concentration of AA >24.0 ng/ml.

AA: Acetylamantadine.

**Table 4.** Serum concentrations of carcinoembryonic antigen in healthy adult volunteers recruited locally at Dhaka (Bangladesh site).

Subject ID	CEA (ng/ml)	Subject ID	CEA (ng/ml)
H01^†^	2.7	H21	1.9
H02	1.8	H22	1.4
H03^†^	4.1	H23^†^	4.5
H04	0.5	H24	1.3
H05	2.0	H25^†^	4.5
H06^†^	3.3	H26^†^	22.1
H07^†^	3.8	H27	2.3
H08	1.0	H28	1.6
H09	1.5	H29	2.0
H10	1.7	H30^†^	2.9
H11^†^	4.7	H31	1.2
H12^†^	3.4	H32	1.9
H13^†^	5.4	H33	1.7
H14	1.6	H34^†^	2.8
H15	2.1	H35	1.8
H16^†^	3.9	H36	1.2
H17^†^	3.0	H37^†^	2.5
H18^†^	6.1	H38	2.4
H19	1.5	H39^†^	3.1
H20^†^	2.8	H40	2.4

† Identified as high CEA levels with ≥2.5 ng/ml as the cutoff.

**Table 5.** Follow-up amantadine test in the outlier group at Dhaka (Bangladesh site).

Subject ID	Initial			Follow-up 1			Follow-up 2		
	2 h [AA] (ng/ml)	4 h [AA] (ng/ml)	6 h [AA] (ng/ml)	2 h [AA] (ng/ml)	4 h [AA] (ng/ml)	6 h [AA] (ng/ml)	2 h [AA] (ng/ml)	4 h [AA] (ng/ml)	6 h [AA] (ng/ml)
H03	11.9	165	156	21.0	30.3	41.4	11.4	113	30.9
H07	11.0	29.1	66.4	28.4	60.2	64.7	1.7	33.4	19.5
H11	3.0	185	45.1	7.1	36.3	77.2	1.5	45.2	67.3
H12	31.4	132	91.3	92.6	81.5	46.5	46.1	120	54.9
H17	9.8	60.8	27.3	39.4	71.2	42.3	6.7	25.0	48.3
H18	22.8	28.2	25.1	17.5	74.9	103	5.9	19.7	9.3
H26	14.9	24.0	8.0	6.8	46.9	21.5	1.8	14.3	26.4

Urinary AA concentrations were determined at 2, 4 and 6 h postamantadine ingestion at first follow-up (6 month) and at second follow-up (9-month) after the initial amantadine test.

AA: Acetylamantadine.

In order to understand the significance of the higher AA concentrations observed at the 6-month follow-up, a clinical assessment was conducted. In the two males in this follow-up cohort (H07 and H26), ultrasound revealed enlarged prostates with normal prostate-specific antigen values. With respect to the clinical features in the female outliers, mammogram revealed fibrotic changes in both breasts (Table 6). In addition, ultrasound also showed the presence of hepatomegaly and fatty changes in the liver in all seven individuals. Hematological assessments showed either elevated white blood cell count (H07 and H12) or elevated platelets (H11) or a platelet count in the upper range of normal (H17).

**Table 6.** Clinical features of the healthy adult volunteer outlier group at Dhaka (Bangladesh site).

Subject ID	Sex (M/F)	Age (years)	Mammogram/ultrasound	Hematology (cells/μl)
H03	F	44	Enlarged axillary LN, mild fibrotic change in both breasts; mild hepatomegaly with fatty change; cholelithiasis	Normal
H07	M	42	Mildly enlarged prostate (PSA normal); mild fatty change in liver	Elevated WBC (15,000)
H11	F	56	Mild fibrotic change in both breasts; mild hepatomegaly with fatty change	Elevated platelets (450,000)
H12	F	34	Underlying mass lesion, dense breasts; mild fatty change in liver	Elevated WBC (12,600)
H17	F	38	Mild fibrotic change in both breasts; mild fatty change in liver	High platelets (420,000), eosinophilia
H18	F	52	Mild fibrotic change in both breasts; mild fatty change in liver	Normal
H26	M	57	Upper limit of normal prostate (PSA normal); mild hepatomegaly with fatty change	Normal

Normal range for WBCs, 4500–11,000 WBC/μl of blood; normal range for platelets 150,000–450,000 platelets per μl of blood.

F: Female; LN: Lymph node; M: Male; WBC: White blood cell.

#### Urinary concentrations of AA in healthy adult volunteers (Winnipeg site)

We conducted a follow-up with the Winnipeg outliers within the healthy adult group. In this study, 11/20 (55%) participants were considered as outliers, in other words, having a higher than expected urinary concentration (≥6.0 ng/ml) or total amount (>600 ng) of AA at 4 h (Table 7). These individuals were followed-up with a health questionnaire asking if they had experienced any health issue since completion of the study. On the basis of the responses to the questionnaire as well as accessing electronic medical records (CancerCare Manitoba), three of these outliers experienced some medical issue related to cancer, whereas one participant had registered with CancerCare Manitoba, but there was no diagnosis or clinical information available (Table 8).

**Table 7.** Urinary acetylamantadine concentration in healthy adult volunteers recruited locally at Asper Clinical Research Institute (Winnipeg site).

Subject ID	2 h [AA] (ng/ml)	Urine (ml)	Total AA (ng)	4 h [AA] (ng/ml)	Urine (ml)	Total AA (ng)	6 h [AA] (ng/ml)	Urine (ml)	Total AA (ng)
BM0001	2.0	220	429	2.2	205	445	3.5	130	452
BM0002^†^	10.5	20	210	10.6	40	424	10.7	75	803
BM0003^†^	1.7	180	297	3.4	238	809	1.4	228	309
BM0004^†^	1.1	410	442	2.8	320	880	4.5	130	589
BM0005^†^	3.8	40	152	3.2	190	614	1.4	180	252
BM0006^†^	1.2	85	99	8.7	145	1260	2.0	165	333
BM0007^†^	3.0	165	501	14.9	120	1788	4.0	175	707
BM0008	1.6	190	296	1.7	205	344	5.0	170	841
BM0009^†^	3.9	170	660	9.2	130	1191	5.8	210	1210
BM0010	1.1	7	8	0.8	120	99	0.4	100	42
BM0011^†^	0.8	410	314	3.2	400	1288	1.7	390	655
BM0021^†^	2.7	220	603	9.3	460	4264	5.3	190	1005
BM0022	2.3	90	207	2.0	95	194	2.1	100	211
BM0023	0.6	315	201	3.3	85	282	2.4	155	369
BM0024	4.4	55	239	4.6	90	413	4.4	130	569
BM0025	1.4	50	69	4.0	50	198	2.3	100	229
BM0026^†^	0.6	245	142	2.6	305	790	3.5	75	264
BM0027	5.2	160	837	4.4	80	353	4.6	130	593
BM0028^†^	3.8	220	834	10.7	100	1070	14.8	115	1702
BM0029	4.6	120	550	5.8	100	575	4.5	140	631

† Identified as an outlier based on total AA amount (ng) at 4 h time point of more than 600 ng or concentration of AA more than 6.0 ng/ml.

AA: Acetylamantadine.

**Table 8.** Clinical features of the healthy adult volunteer outlier group at the Asper Clinical Research Institute (Winnipeg site).

Subject ID	Sex (M/F)	Age (years)	Follow-up questionnaire/electronic medical records
BM0021	M	50	No health issue
BM0026	M	43	No health issue
BM0028	M	18	No health issue
BM0002	F	24	No health issue
BM0003	F	57	Thyroid nodules (2013)
BM0004	F	34	Ovarian cancer stage 1C (2014)
BM0005	F	49	Lung nodules NYD (2016)
BM0006	F	46	Registered with CCMB, but no diagnosis or information currently available
BM0007	F	28	No health issue
BM0009	F	22	No health issue
BM0011	F	25	No health issue

CCMB: CancerCare Manitoba; NYD: Stage not yet determined.

## Discussion

We have recently reported that human cancer is associated with high urinary concentration of AA [20,21] with receiver-operating characteristic (ROC) for AA demonstrated to be 0.689 (CI: 0.591–0.786, 95%) in lung cancer and 0.717 (CI: 0.577–0.858, 95%) for breast cancer [21]. In the present preliminary study, the initial data from Bangladesh healthy cohort had a high proportion of ‘outliers’ versus normal AA concentration at 6 h time point (<24.0 ng/ml) or total amount of AA (>2000 ng). Initial analysis of outliers demonstrated a large effect on the mean and median, which in turn affected the error (absolute and mean) in our dataset, and large deviations were observed when the error was plotted. Some of the outliers in Bangladesh had urinary AA concentrations that were between two- and six-times higher than the cutoff points, qualifying them as either intermediate or extreme outliers. This important deviation suggested that there could be valuable information to obtain and thus the ‘outliers’ were analyzed separately to try to understand the significance and clinical relevance of the higher AA concentrations. Also, in view of the small outlier sample size in both the Bangladesh versus Winnipeg cohorts it was not possible to conduct sensitivity analyses.

Since there was no apparent measurement or experimental design that would have led to exclusion of the outliers, we commissioned an assessment and follow-up to determine the health and qualification of status over a period of time. Accordingly, a thorough examination of the outliers at 6- and 9-month follow-up that entailed the amantadine test and comprehensive clinical and hematological assessments were conducted at the Bangladesh site. This leads to a follow-up study with the Winnipeg cohort, but this entailed a questionnaire as well as accessing electronic medical records; however, the ‘outliers’ in this cohort were outside of normal AA concentration (<6.0 ng/ml) or total amount of more than 600 ng at the 4 h time point.

The follow-up analysis revealed that the amantadine test is possibly detecting cancer at a very early stage; therefore, it could be useful in screening populations at high risk for cancer. It should be noted that while a further increase in AA concentration in the 6-month follow-up compared with values in the first amantadine test was observed in some of Bangladesh outliers, a reduction in AA concentration at the 9-month follow-up was observed. These values were almost comparable to the AA concentration observed in the initial amantadine test. This biphasic nature of the AA concentrations are suggestive that the increase in AA serves as the trigger in the initiation of the processes involved in cell proliferation and growth, while the subsequent reduction in AA can be seen as an adaptive mechanism.

It should be mentioned that follow-up clinical assessment at the Bangladesh site, seven outliers had developed hepatomegaly and/or fatty liver. Hepatomegaly can occur as a consequence of infection, metabolic disorders, congestive heart failure or hepatic tumor [24–26]. Interestingly, high rates of liver cancer are known to occur in areas with high contamination levels of the carcinogen arsenic, such as in Bangladesh [27–29]. Indeed most of the participants recruited into the study as healthy subjects resided in areas with moderate to severe arsenic contamination [30] (based on recorded area of residence on screening forms [data not shown]). Furthermore, individuals with severe fatty liver (non-alcoholic fatty liver disease) need to be monitored for liver cancer because of the link between fatty liver disease and liver cancer (hepatocellular carcinoma) [31,32]. It is pointed out that the connection between arsenic and cancer has been studied in some regions of Chile, where arsenic contamination is considered to be the primary cause of mortality due to lung cancer that is more than threefold as compared with noncontaminated areas in the same country [33].

While elevated white blood cell count is indicative of an infection, it has also been linked to other inflammations and in particular cancers gastric, lung and blood cancers [34–36]. Two of the outliers (H11 and H17) showed an elevated platelet count and eosinophilia, respectively. Eosinophilia has been linked to a wide variety of non-neoplastic disorders, as well as to neoplastic conditions [37] and an elevation in eosinophils may exert protumor effects [38,39]. On the other hand, inflammation and platelet activation at the site of tissue damage is known to contribute to initiate a cascade of events, which promote tumorigenesis [40]. In fact, platelets release a wide variety of proteins, including growth and angiogenic factors, lipids, and extracellular vesicles rich in genetic material, which can mediate the induction of phenotypic changes in cancer cells, promoting carcinogenesis and metastasis [40].

Although being underweight has been linked to an increase in the risk of adverse health complications, overweight/obesity increases the risk of developing several cancers [41–43]. With respect to our observations, five outliers in the Bangladesh healthy group that had some pathophysiological indication of presymptomatic cancer (Table 6), two of them were classified as obese (H11, H18; BMI >30.0) and two other were deemed as overweight (H17, H26; BMI 25.0–29.9). Similarly, two-third outliers in the Winnipeg healthy group (Table 2) were classified as overweight (BM0003, BM0004). Unfortunately for the one participant that was classified as obese (BM0006) and registered at CancerCare Manitoba, no clinically relevant information was available. Taken together, the amantadine test could be of value for assessing risk of cancer in overweight/obese individuals, a possibility that warrants future large trial in this area.

It should also be mentioned that we used different cutoff values for the basal AA concentration in the healthy adults in the Bangladesh versus Winnipeg cohorts. This may be as a consequence of geographical and environmental factors. Indeed, we have previously observed regional/ethnic differences in AA concentration in healthy adults [44] that may be a reflection of the influence of environmental, socioeconomic and lifestyle factors affecting the basal SSAT-1 activity. However, these factors remain incompletely understood warranting further investigation. In addition, while not recorded in the present study, in subsequent studies, family history of cancer should be documented as it is conceivable that the amantadine test may be detecting presymptomatic cancer in populations that may be genetically predisposed for cancer.

Current standards for determining and verifying the presence of cancer involve computed tomography, MRI and ultrasound in conjunction with molecular and protein biomarkers; however, their use is limited because of the challenges regarding costs, accessibility, exposure to ionizing radiation and levels of false-positive results. In addition, biopsies for cancer diagnosis are invasive, time consuming, distressing, expensive and cannot be performed repeatedly [10,45]. Thus, the simple, noninvasive, no risk, painless and cost-effective amantadine test may become an alternative or supplementary assessment tool to identify and follow malignant disease. Furthermore, with this test there is no need for surgery and the potential to reduce diagnosis needs to be further examined.

## Conclusion

Analysis of the ‘outliers’ observed in previous studies using the amantadine test revealed that a large number were at risk or developed cancer, suggesting that the test can predict the occurrence of cancer. Monitoring of urinary AA concentration combined with clinical and hematological characteristics could be established as a useful tool for purposes of screening and follow-up for cancer in high-risk populations.

## Future perspective

Biomarkers that detect cancer and monitor response to treatment will be highly beneficial. Our findings in ‘outliers’ support usefulness of amantadine as a screening or surveillance test in populations considered at high risk for developing cancer. It is also possible that this test could be used for monitoring patients after curative surgical or chemoradiation therapy to assess eradication of the tumor. Also, in follow-up, it could possibly detect proliferation of new cancer cells (relapse).

**Summary points**

- We have previously reported that increased spermidine/spermine N^1^-acetyltransferase activity is linked to cancer.
- In the present study, a higher urinary concentrations of acetylamantadine were measured in some of the healthy adult volunteers at both study sites.
- A follow-up of the outliers that entailed hematological assessments, mammogram, ultrasound as well as additional amantadine test was carried out with the Bangladesh group. A health questionnaire and access to electronic medical records was performed with the Winnipeg site outliers.
- The follow-up data obtained at both sites are suggestive that the amantadine test may be detecting cancer cases prior to clinical symptoms.
- The amantadine test could potentially serve as a novel, simple to use and low-cost early detection test for cancer or for surveillance in populations considered at high risk for cancer.
- These possibilities warrant future large-scale studies.
